# Low Level of Perfectionism as a Possible Risk Factor for Suicide in Adolescents With Attention-Deficit/Hyperactivity Disorder

**DOI:** 10.3389/fpsyt.2021.707831

**Published:** 2021-09-13

**Authors:** Luca Katzenmajer-Pump, Bernadett Frida Farkas, Balázs András Varga, Johan M. Jansma, Judit Balázs

**Affiliations:** ^1^Institute of Psychology, Eötvös Loránd University, Doctoral School of Psychology, Budapest, Hungary; ^2^Doctoral School of Mental Health Sciences, Semmelweis University, Budapest, Hungary; ^3^Brain Center Rudolf Magnus, Department of Neurology and Neurosurgery, UMC Utrecht, Utrecht, Netherlands; ^4^Department of Developmental and Clinical Child Psychology, Institute of Psychology, Eötvös Loránd University, Budapest, Hungary; ^5^Department of Psychology, Bjørknes University College, Oslo, Norway

**Keywords:** ADHD, perfectionism, association, suicidal behavior (SB), riskfactor

## Abstract

**Introduction:** Suicide is one of the leading causes of death among adolescents. Although it is known that both perfectionism and attention-deficit/hyperactivity disorder (‘ADHD’) are important risk factors for suicide, there are no studies that have investigated the relationship between suicidal behavior and perfectionism among people with ADHD.

**Aim:** The current study investigates the association between perfectionism and suicide in adolescents with ADHD.

**Method:** Subjects included 88 adolescents with ADHD and 96 non-clinical control adolescents. We used the Multidimensional Perfectionism Scale to evaluate perfectionism as well as its separate traits, and the Mini International Neuropsychologic Interview Kid to evaluate psychiatric disorders and suicidal behavior. Differences between the groups were statistically evaluated using *t*-tests, a Poisson regression analysis with suicide as a discrete variable, and a logistic regression analysis with suicide as a binary variable.

**Results:** Compared to the control group, the ADHD group showed a significantly lower level on the adaptive ‘Organization’ trait of perfectionism, but not on any other trait, and a significantly higher level of suicidal behavior.

Logistic regression results indicated a significant association for perfectionism in general (OR = 0.93, *p* = 0.003), and for the ‘Personal Standards’ trait (OR: 0.82, *p* = 0.039).

Poisson regression analysis also showed a significant association with perfectionism in general (IRR = 0.90; *p* < 0.001) and with the ‘Personal standards’ trait model (IRR = 0.81, *p* = 0.019).

**Discussion:** Our results indicate that a low level of perfectionism, in particular ‘Personal standards’, may be a risk factor for suicidal behavior in adolescents with ADHD. We recommend that psychoeducation and therapy of adolescents with ADHD should consider focusing on adaptive perfection as a possible risk factor for suicide as well.

## Introduction

Attention-Deficit/Hyperactivity Disorder (‘ADHD’) is one of the most diagnosed psychiatric disorder among children and adolescents (1), with a prevalence of 4–6% in children, while up to two-thirds of them have impairments in adulthood as well (2–4). The core symptoms of ADHD include inattention, hyperactivity and impulsivity (American Psychiatric Association, 2013). People with ADHD often endure stigmatization due to their symptoms including social rejection (5–7). According to Wu (8) ADHD is associated with poorer school tests, and they drop out of school more often. Faraone (9) found that children with ADHD have more often learning disabilities, are more often placed in special classes and perform poorly in classroom activities. Moreover, Kent (10) found that, even if controlled for IQ, there was a significant difference in cumulative grade point averages between adolescent males with ADHD and non-clinical adolescent males.

Both impulsivity and perfectionism (11) are transdiagnostic personality factors which previously were thought to exclude each other because impulsivity is characterized by a lack of control, while perfectionism is characterized by a rigid need for control. However, recent studies have suggested that perfectionism may play a more important role in ADHD than previously thought. It has been shown for instance that both impulsivity and perfectionism can be related to externalizing and internalizing pathology disorders and thus they do not necessarily exclude each other (12). Indeed, there have been several recent studies that have indicated a connection between perfectionism and ADHD: Strohmeier et al. (13) found that perfectionism was predominantly associated with adult ADHD and was associated with procrastination and delaying the start of a task until all conditions are fulfilled (13). Greven et al. (14) found that the severity of ADHD symptoms was associated with an increase in internalizing and externalizing problems as well as an increase in perfectionism. Arancibia et al. (15) found that so-called ‘cluster C’ personality traits such as perfectionism, dependency and anxiety were significantly associated with ADHD.

Importantly, there is also a growing body of research evidence that both ADHD is associated with an increased risk of suicide, which is a leading cause of death among adolescents [e.g., (1, 16)]. According to a systematic review of Balazs and Kereszteny (1), there is a positive association between ADHD and suicidal behavior both in children and adolescents. A second longitudinal review by Garas and Balazs (17) which investigated suicide risk in ADHD patients found a positive association between ADHD and the presence of suicidal behavior at follow-up visits. Additionally, Manor et al. (18) investigated 23 subjects, who were admitted to the emergency room as a result of an attempted suicide and found that 15 did meet the criteria for an ADHD diagnosis, while only five of them were actually diagnosed with ADHD.

In addition to this, several studies have also indicated a positive relationship between perfectionism and suicidal behavior (16, 19–21) such as Hamilton and Schweitzer (19) found that perfectionism was associated with an increased ‘vulnerability to suicide ideation’ among university students. Limburg et al. (16) performed a meta-analysis focusing on perfectionism and psychopathology and found that perfectionism was strongly linked to depression, obsessive-compulsive disorder and anxiety disorder. Recently, Abdollahi and Carlbring (20) focused on different aspects of perfectionism regarding suicidal ideation: while ‘neurotic perfectionism’ was positively associated with suicidal ideation, ‘adaptive perfectionism’ was negatively associated with suicidal ideation. A meta-analysis, published by Smith et al. (21), found that striving for perfection was associated with an increase in suicidal ideation while the ‘Organization’ dimension of perfectionism was associated with a reduced level of suicide ideation. Other traits of perfectionism did not affect suicidal ideation.

Based on these recent new insights, the main aim of the present study was to explore if perfectionism may be a risk factor for suicidal behavior in adolescents with ADHD. We believe that increased knowledge in this area may help to improve suicide prevention in patients with ADHD.

## Materials and Methods

### Participants and Procedure

The study was conducted with the approval of the Ethical Committee of the Medical Research Council, Hungary (ETT-TUKEB, project identification code: 50922-2/2017/EKU). Subjects were provided with written informed consents after being verbally informed on the nature of the study. After signing the informed consent, a diagnostic interview was recorded by a psychologist (see below). Afterwards, self-rated questionnaires were completed by all subjects. No compensation was provided to the participants.

Adolescents with ADHD were recruited from the Vadaskert Child Psychiatric Hospital and Outpatient Clinic, Budapest, Hungary, in Szent Rokus Hospital and Outpatient Clinic, Baja and Tolna County's Balassa Janos Hospital and Outpatient Clinic, Szekszard. Control group participants were recruited from several high schools in Hungary. Both groups included subjects between 13 and 18 years. Further inclusion criteria for the clinical group included an ADHD diagnosis from a psychiatrist which needed to be confirmed by a structured psychiatric diagnostic interview (see below).

Exclusion criterion for both the ADHD and control groups included a medical history of intellectual disability. Further exclusion criteria for the control group included: current or previous psychiatric diagnoses or psychological treatment (see below).

### Questionnaires

#### Mini International Neuropsychologic Interview for Children and Adolescents

Psychiatric symptoms and disorders were diagnosed using the modified version of the Mini International Neuropsychologic Interview for Children and Adolescents (MINI Kid). The modified version of the MINI Kid evaluates the presence of twenty-four psychiatric diagnoses according to the Diagnostic and Statistical Manual of Mental Disorders fifth edition (DSM-5) (APA, 2013), including ADHD and their subthreshold forms and suicidal behavior (22, 23). The original English language MINI Kid has been shown to have adequate validity and reliability (22). Balázs et al. (23) developed a Hungarian version of the MINI Kid, which has also been shown to have adequate test-retest reliability, specificity, and sensitivity (23). The questions of the MINI Kid follow the DSM-5 criteria of the disorders. In its original form, the structure of the MINI Kid is branching, which means, if the core symptoms of a disorder are not present, the additional questions on the symptoms of that disorder should not be asked. However, in the current study we used the modified version of the MINI Kid, which means we excluded the ‘branching logic’ and in this way the MINI Kid evaluated all the possible symptoms of a disorder.

Current suicidal behavior was assessed via the following questions of the MINI Kid: “In the past month did you” “Wish you were dead?”, “Want to hurt yourself?”, “Think about killing yourself?”, “Think of a way to kill yourself?”, “Attempt suicide?” Have a method in mind to kill yourself?” Participants met the criterion for suicidal behavior after one “yes” answer and according to the points, suicidal behavior level was either “low” or “moderate” or “high” (22, 23). The MINI Kid was administered by interviewees who were MA or PhD students in psychology or medicine. To ensure validity and inter-rater reliability, all interviewers took part in a training course on the MINI Kid before the study and were under continuous supervision by the leader of the research. As all participants were older than 13, they were interviewed according to the instructions of the Mini Kid administration procedure without parental participation (22, 23).

#### Multidimensional Perfectionism Scale

The level of perfectionism was evaluated with the ‘Multidimensional Perfectionism Scale’ (‘FMPS’), a self-report scale developed by Frost et al. (24): It contains 35 questions, which are grouped into four ‘maladaptive’ and two ‘adaptive’ traits of perfectionism (24–26).

Maladaptive traits cause a negative reaction if standards are not reached and include: ‘Concern Over Mistakes’ (reflecting negative reactions to errors), ‘Parental Criticism’ (the perception of parents being too critical), ‘Parental Expectation’ (the perception of high parental standards), ‘Doubt About Actions’ (the tendency to doubt abilities in actions). Adaptive traits do not evoke negative reactions if the goal is not reached and include: ‘Personal Standards’ (setting high standards for assessments), ‘Organization’ (the ability and importance placed on orderliness).

Response method used a 5-point liker scale (1 = strongly disagree and 5 = strongly agree). The internal consistency of the subscales ranged from 0.77 to 0.93 (26). The total score of the FMPS is calculated by summing the scores of each trait except ‘Organization’ (24–26). According to Frost et al. (24) the ‘Organization’ trait showed a low correlation with other total traits of the perfectionism scale, therefore it was suggested to leave this trait out when looking at the total score (24). The FMPS scores are strongly correlated with scores of other questionnaires of perfectionism (r = 0.85 with Burns Perfectionism Scale, and r = 0.59 with the Perfectionism Scale from the Eating Disorder Scale) (24).

#### Demographic Questionnaire

A structured parent-rated questionnaire was developed specifically for this study to gather information about the demographic data of the participants, including the combined education level of the parents, family history of psychological or psychiatric treatment, economic activity of the father/mother (e.g. active worker, unemployed, retired, full-time parent, deceased), as well as the structure of the family. The questionnaire consisted of closed questions and each answer belonged to a point.

### Statistical Analysis

#### Comparison of Levels of Perfectionism and Suicidal Behavior

Statistical analyses were performed using Stata 14 (27). Differences in FMPS scores between the ADHD and control group were evaluated using *t*-tests. The distribution of the suicide scale of the MINI Kid significantly violated normality assumptions (28) and was therefore evaluated using a non-parametric Mann-Whitney test and a Spearman correlation test. Because multiple comparisons were performed, the *p*-value for each variable was calculated using a Holm-Bonferroni correction method (adjusted *p*-value). Effect sizes were calculated with Cohen's d for the parametric test (*t*-test), a rank correlation r- value for the non-parametric Mann-Whitney test (29) and the Cramer's V for comparisons between two nominal variables. For all descriptive statistics, the reliability was above.70 and thus acceptable (30). The descriptive statistics of the variables can be found in Table 1.

**Table 1:** The descriptive statistics of the variables.

	**ADHD**					**Non**-**clinical controls**				
**Variable**	***N***	**Mean**	**SD**	**Min**	**Max**	***N***	**Mean**	**SD**	**Min**	**Max**
Age	88	14.6	1.77	13	18	96	14.8	1.53	13	18
Parental education	88	3.05	1.78	0	6	96	4.09	1.13	2	6
Concern over mistakes(perfectionism)	88	20.8	7.17	9	42	96	20.9	7.29	9	39
Personal standards(perfectionism)	88	18.6	6.32	7	35	96	20.2	6.15	8	34
Parental expectations(perfectionism)	88	12.2	4.52	5	25	96	12.0	4.23	5	20
Parental criticism(perfectionism)	88	8.77	3.89	0	18	96	8.12	3.47	4	20
Doubt about actions(perfectionism)	88	10.9	3.61	4	20	96	10.1	3.60	4	18
Organization(perfectionism)	88	21.7	5.19	6	30	96	24.0	4.70	11	30
General scale(Perfectionism)	88	71.3	20.0	35	137	96	71.2	19.1	32	121
Suicidal symptom scale	86	0.30	1.17	0	7	96	0.01	0.10	0	1

*ADHD, attention-deficit/hyperactivity disorder; SD, standard deviation*.

#### Association Between Perfectionism and Suicidal Behavior in ADHD

Two types of regression models were used to analyze suicidal behavior as we believe they both provide important information on the phenomenon. In the first clinical approach, suicidal behavior is included as a binary variable representing the presence of the symptom and evaluated using a logistic regression approach. For this approach we calculated the Odds Ratio (‘OR’) as a relative measure of association. The OR is the ratio of the odds of an event occurring in one group to the odds of it occurring in another group.

For the second approach, suicidal behavior was evaluated as a continuous discrete variable and evaluated using a Poisson regression. For this approach, the Incidence Rate Ratio (‘IRR’) was calculated as a measure of association.

Within this analysis, over dispersion was evaluated using the alpha statistic. If standard deviations were not equal (indicated by an alpha significantly greater than zero), a negative binomial regression was used instead of a Poisson regression (31).

For both approaches, we evaluated perfectionism either as a unidimensional or multidimensional concept. The unidimensional approach includes only one total score of perfection, based on all traits except ‘Organization’. For the multidimensional approach we included all traits of perfection (except ‘Organization’) as separate variables.

The other explanatory variable included in each regression analyses was a combined ADHD diagnosis. The two ADHD variables (‘Predominantly Inattentive’, ‘Predominantly Hyperactive/Impulsive’) were combined into one variable because of their high dependence [ρ_(182)_ = 0.69, *p* < 0.001].

Control variables for all regression analyses included age and the education level of the parents. Gender was not included as a control variable because the ADHD group was too severely skewed toward male subjects (92% male). The best model fits were decided by using the Akaike information criterion (‘AIC’) index. If the control variables did not provide a better fit for the models, then they were not included in the regression analysis.

## Results

### Sample

In the present study the ADHD group included 88 adolescents (mean age: 14.58; SD: 1.77) and the control group 96 adolescents (mean: 14.77; SD: 1.53). There was no significant difference in age between the groups. In regard to gender, there was a significant difference between the clinical and control group: in the control group 46% of the participants were girls whereas in the clinical group only 8% of the participants were girls [χ2_(1)_ = 39.32; *p* < 0.0001, *V* = 0.46].

Table 2 presents the comparison of ADHD and control groups regarding information about the demographic data of the participants.

**Table 2:** Comparison of ADHD and control groups regarding information about the demographic data of the participants.

		**ADHD** ***n*** **= 88**	**Nonclinical** **controls** ***n*** **= 96**
Combined education level of the parent scoring: 1 = High School; 2 = College; 3 and above = University		3.05 (SD=1.78)	4.09 (SD=1.13)
Family history of psychological or psychiatric treatment *n* (%)	Yes No	50 (59.52%) 34 (40.48%)	45(47.87%) 49 (52.13%)
Father's economic activity *n* (%)	Active worker	57 (80.28%)	81 (84.38%)
	Unemployed	6 (8.45%)	2 (2.08%)
	Retired	3 (4.23%)	3 (3.13%)
	Full-time parent	1 (1.41%)	6 (6.25%)
	Deceased	4 (5.63%)	4 (4.17%)
Mother's economic activity *n* (%)	Active worker	47 (62.67%)	75 (78.95%)
	Unemployed	10 (13.33%)	4 (4.21%)
	Retired	2 (2.67%)	1 (1.05%)
	Childcare	1 (1.33%)	7 (7.37%)
	Full-time parent	14 (18.67%)	7 (7.37%)
	Dependent	1 (1.33%)	0 (0.00%)
	Deceased	0 (0.00%)	1 (1.05%)
The structure of the family *n* (%)	Original full family	41 (50.62%)	72 (75.79%)
	Incomplete family (mother only)	19 (23.46%)	11 (11.58%)
	Incomplete family (father only)	0 (0.00%)	1 (1.05%)
	Complete family with foster mother	5 (6.17%)	2 (2.11%)
	Complete family with foster father	13 (16.05%)	9 (9.47%)
	Family with other relative	3 (3.70%)	0 (0.00%)

*ADHD, attention-deficit/hyperactivity disorder; SD, standard deviation. No data available of the 88 ADHD participants to the question: ‘Family history of psychological or psychiatric treatment’: n = 4 ‘Father’s economic activity’: n = 17, ‘Mother's economic activity’: n = 13, ‘The structure of the family’: n = 7. No data available of the 96 Nonclinical controls: ‘Family history of psychological or psychiatric treatment’: n = 2, ‘Father's economic activity’: n = 0, ‘Mother's economic activity’: n = 1, ‘The structure of the family’: n = 1*.

### Comparison of Perfectionism Between the ADHD and Control Group

The comparison of the ADHD and control group regarding each perfectionism scale can be found in Table 3. There was one subscale showing a significant difference between the groups, namely ‘Organization’: The ADHD group scored significantly lower on this trait than the control group [t_(183)_ = 3.153 *p* = 0.002]; effect size medium [*d* = 0.47; clinical group: *M* = 21.73; sd = 5.18, control group: *M* = 24.02; sd: = 4.7, standard deviations were homogeneous (*F* = 0.946; *p* = 0.332)]

**Table 3:** Comparison of ADHD and controls regarding suicidal behavior and perfectionism.

**Variable**	**Significance test** **ADHD—control**	**Adjusted** ***p*** **value**
Concern over mistakes	t_(183)_ = 0.095	1.00
Personal standards	t_(183)_ = 1.68	0.76
Parental expectations	t_(183)_ = −0.26	1.00
Parental criticism	t_(183)_ = −1.18	1.00
Doubt about actions	t_(183)_ = −1.38	1.00
Organization^*^	d: 0.47 t_(183)_ = 3.15	0.016
Perfectionism (general)	t_(183)_ = 0.04	1.00
Suicidal behavior	*R* = 0.22 z(181) = −2.98	0.016

*Adjusted p value: calculated for each variable, using a Holm-Bonferroni correction method*.

### Comparison of Suicidal Behavior Between the ADHD and Control Group

Table 3 presents the comparison of ADHD and control groups regarding suicidal behavior and perfectionism. The distribution of the suicide scale (symptom frequency) was significantly skewed due to the rarity of the symptoms, therefore a non-parametric Mann-Whitney analysis was applied, as mentioned in the methods section. Statistical evaluation of the suicide scale revealed a significantly higher presence of suicidal behavior in the ADHD group, but the effect size was low [z_(182)_ = −2.975, *p* = 0.003, *r* = 0.22].

### Negative Binominal Regression

Negative binominal regression analysis used suicidal behavior as a discrete variable. According to the AIC index, the model without demographic variables showed the best fit.

For the simple model, the IRR for the variable ADHD was 1.57 (*p* – 0.012), indicating that if the combined ADHD scale increased by one unit, the incident rate for suicidal behavior increases with 57 %. The IRR for ‘Total Perfectionism’ was 0.90, (*p* < 0.001), which indicates that if the perfectionism scale increases by one point, it would reduce the incident rate of suicide behavior scale with 10%. The detailed results of the simple model regression can be found in Table 4.

**Table 4:** Negative binominal regression analysis with discrete suicide variable.

**Suicide scale**	**IRR**	**st. err**.	***z*** **-value**	***p*** **-value**	**95% conf. int**.
Perfectionism (general)^**^	0.90	0.025	−3.75	<0.001	0.85–0.95
ADHD (combined)^*^	1.57	0.28	2.50	0.012	1.10–2.24
Other statistical values					
Mean dependent var	0.15	SD dependent var		0.82	
Pseudo r-squared	0.18	Number of obs		182	
Chi-square	21.1	Prob > chi2		<0.001	
Akaike crit. (AIC)	104.5	Bayesian crit.		117.3	

*ADHD, attention-deficit/hyperactivity disorder; IRR, incidence rate ratio; st.err, standards error; 95 conf.int: confidence interval*.

The extended model revealed a significant association for ‘Personal standards’ (IRR = 0.81, *p* = 0.019), indicating that for each unit increase in the Likert-scale for ‘Personal Standards’, the percent change in the incident rate of suicidal behavior was 19%.

### Logistic Regression Analysis Results

The logistic regression approach used suicide as a binary variable. According to the AIC indicator, the fit of the model without demographic variables showed the best fit.

The simple model indicated a significant association for ‘total perfectionism’ (OR = 0.93, *p* = 0.003), indicating that an increase in one unit on the perfectionism score reduced the chance of suicidal behavior by 7%. The presence of ADHD increased the chance of suicidal behavior by 48% (OR = 1.48, *p* = 0.012). Table 5 shows the detailed results for the simple model.

**Table 5:** Logistic regression results with binary suicide variable.

**Dummy**	**OR**	**st.err**.	***z*** **-value**	***p*** **-value**	**95% conf. int**.
Perfectionism (general)^**^	0.93	0.023	−3.02	0.003	0.88–0.97
ADHD^*^ (combined)	1.48	0.23	2.50	0.012	1.09–2.00
Other statistical values					
Mean dependent var	0.060	SD dependent var		0.24	
Pseudo r-squared	0.203	Number of obs		182	
Chi-square	16.9	Prob > chi2		<0.001	
Akaike crit. (AIC)	72.2	Bayesian crit.		81.8	

** *p <0.01*,

* *p < 0.05. ADHD, attention-deficit/hyperactivity disorder; OR, odds ratio; st. err, standards error; 95% conf.int: confidence interval*.

With the extended model including five perfectionism traits, the factor ‘Personal Standards’ was significantly associated with suicide behavior [OR: 0.82 (*p* = 0.039)]. This result indicates that an increase of one unit in ‘Personal Standards’ reduced the chance of suicidal behavior by approximately 8%.

## Discussion

According to our knowledge this is the first study which investigates if perfectionism is a risk factor for suicidal behavior in adolescents with ADHD. The main finding of the current study is that the level of suicidal behavior was negatively associated with adaptive perfectionism.

Our study also indicated that adolescents with ADHD show a significantly higher level of suicidality than the control group. This result is in line with previous studies which have also indicated a significantly increased level of suicidality in adolescents with ADHD (1, 11, 32, 33).

We found a significant *negative* association between adaptive perfectionism and suicidal behavior among adolescents with ADHD. This result is in line with several previous findings in clinical populations with other psychiatric disorders [e.g., (34–36)]. Only the adaptive ‘Personal Standards’ trait was found to be significant in the perfectionism model by having a negative association with suicidal behavior among adolescents with ADHD. Moreover ‘Personal Standards’ may indeed be a protective factor for suicide in an ADHD population. These results have drawn the attention of clinicians to the importance of improving adaptive perfectionism in patients with ADHD. The study of Abdollahi et al. (37) provides compelling evidence for the importance of ‘cognitive behavioral therapy’ (‘CBT’) in the reduction of maladaptive perfectionism in patients with social anxiety disorder (‘SAD’). Further studies that focus on the efficacy of CBT in increasing the level of adaptive perfectionism in patients with ADHD, could help improve prevention of suicide in this population. As our study is the first to investigate the role of perfectionism as a possible risk factor for suicide in adolescents with ADHD, our results need to be replicated by future studies.

Besides our main findings, our results also indicated that within the subscales of perfectionism, adolescents with ADHD only scored lower than nonclinical controls on the adaptive ‘Organization’ trait. Over the past 30 years, a large body of research has examined the role of perfectionism in a variety of psychiatric disorders, including anxiety disorders, depression, eating disorders, suicide risk, social phobia (38–41) as well as ADHD (13–15). While Frost et al. (24) stated that perfectionism is linked to a higher level of psychopathology, there have been studies that have contradicted this statement (38–41). Our result is in line with previous studies: Weyandt and DuPaul (42) found that adolescents with ADHD have poorer organizational skills and a recent study by Christian et, al. (12), using latent profile analysis to identify subgroups in a population of 1,353 undergraduate students, found that the dimensions ‘impulsivity’ and ‘perfectionism’ can co-occur within the same person. Stoeber and Otto (36) already raised concerns whether ‘Organization’ is part of the perfectionism construct (24) and previously considered ‘Organization’ to be associated with perfectionism, but not a defining trait. Thus, it is not impossible that adolescents with ADHD have low organizational skills in combination with a normal level of perfectionism.

Based on these results the psychoeducation and therapy of adolescents with ADHD should focus on their organizational skills as well. Our results underline the importance of Buitelaar et al. (33) review on the treatment strategies for children and adolescents with ADHD, which suggests that these strategies should include time management, planning, and organizational skills. Bul et al. (43) invented a gaming approach specifically for children and adolescents for ADHD focused on the improvement of time management, organizational and planning skills. Langberg et al. (44) found that teachers’ and parents’ ratings of material management was the best predictor of cumulative grade. They suggested that organization is critical when adolescents with ADHD are completing their homework. Wiener et al. (33) found that if teachers had knowledge about an ADHD diagnosis, it increased the understanding of the organizational problems.

Our results should be interpreted in the light of the following limitations: First, it is a cross-sectional study, we were not able to assess any causational relationships. Second, we used self-rating scales for the assessment of perfectionism. It is always a chance by the self-rated scales of not marking the most relevant answer by rushing through the questions or making themselves seems better. Third, there was a gender difference between the ADHD and control groups, however it is important to note that this difference does reflect the natural occurrence of ADHD, as ADHD is more common among boys than girls (45). Fourth, there are no published norms of the FMPS, but several normative information can be found in the various articles published using the scale (24, 25, 45–52).

In conclusion, our study is the first to report that perfectionism, in particular ‘Personal standards’, was associated with an increase in suicidal behavior in adolescents with ADHD. This result suggests that a low level of adaptive perfectionism may be a risk factor for suicidal behavior in adolescents with ADHD. Our findings could have important clinical implications, as this new knowledge could help improve prevention of suicide in this group of patients. Based on these results we recommend that psychoeducation and therapy of adolescents with ADHD should consider focusing on organizational skills as well as adaptive perfection.

## Data Availability Statement

The raw data supporting the conclusions of this article will be made available by the authors, without undue reservation.

## Ethics Statement

The study was conducted with the approval of the Ethical Committee of the Medical Research Council, Hungary (ETT-TUKEB, project identification code: 50922-2/2017/EKU). Each subject as well as their parents provided written informed consents after being verbally informed on the nature of the study.

## Author Contributions

LK-P organized and conducted the data collection and wrote the first drafts of the article. JB was the supervisor of the research and the manuscript process. BF contributed to the data collection, BV conducted the statistical analyses, and JJ made the proofreading of the manuscript and helped in the finalization process. All funders should be credited and all grant numbers should be correctly included in this section. All authors contributed to the article and approved the submitted version.

## Conflict of Interest

The authors declare that the research was conducted in the absence of any commercial or financial relationships that could be construed as a potential conflict of interest.

## Publisher's Note

All claims expressed in this article are solely those of the authors and do not necessarily represent those of their affiliated organizations, or those of the publisher, the editors and the reviewers. Any product that may be evaluated in this article, or claim that may be made by its manufacturer, is not guaranteed or endorsed by the publisher.
